# Aphasia and the Cerebral Speech Mechanism

**Published:** 1898-03

**Authors:** 


**Aphasia and the Cerebral Speech Mechanism.** By William Elder, M.D. Pp. xii., 259. London: H. K. Lewis. 1897.

A difficult subject is here dealt with in an interesting and lucid manner. The reader will find in it a good general account of aphasia, in which both theoretical and pathologico-clinical considerations receive due attention. On the whole there are no important omissions, and the book has the advantage of not being of undue length. This necessitates brevity in some parts of the subject and avoidance of long reports of cases; but the material used is judiciously selected. In dealing with aphasia one has first to consider the theoretical aspects of the question, and then see how far theory is borne out by clinical and pathological data. This plan the author has followed, and with success, in both divisions of the work.

Aphasia is one of the subjects in medicine in which theory founded on psychological data is in advance of observed facts; but such theoretical schemata as are given in this book are valuable, apart from their inherent probability, for the elucidation of unsettled problems, and as a guide to practical observations. In making reports of cases of aphasia it is essential to bear in mind the gaps in our present knowledge, and to examine the patients according to a plan which will leave no important point unanswered. Good directions are here given as to the mode of examination of patients, and if such directions were invariably followed we should rapidly extend our knowledge of aphasia. Meanwhile, the study of cases rather shows that, in the absence of a *post-mortem* examination, deductions as to the nature and extent of the lesion drawn solely from symptoms present during life are apt to mislead.

Dr. Elder takes the views of Broadbent and others with regard to the vexed question of the existence of an ideational centre and of a naming centre. The latter he locates in the posterior half of the 2nd and 3rd temporo-sphenoidal convolutions. He quotes an important case of Mills in favour of this view; but admits at the same time that other necropsies have shown more than one speech centre to be affected. He also follows Wyllie in considering the foot of the 3rd frontal convolution to be a "psycho-motor" or propositionising centre, whilst the lower part of the ascending frontal and parietal convolutions are the seat of centres of vocal or oral articulation only. He reports a case under his own observation where the lesion (hemorrhage) was limited to the lower part of the latter convolutions: the patient had defect in utterance, but no motor aphasia. It seems to us that the hypothesis of an ideational centre is unnecessary, and that the arguments adduced by Bastian in his Lumleian lectures against its existence hold good.

The author make the very pertinent observation that it is just in those cases of aphasia in which there is a limited lesion, and which would be of most value for the elucidation of doubtful problems, that recovery takes place. He gives a case of sensory aphasia of seventeen years' duration, and thinks that a too favourable view has heen taken of the tendency to greater and more rapid recovery in sensory than in motor aphasia, commonly said to be due to a more ready compensation by the right hemisphere. He holds, however, that recovery through compensation by the right hemisphere is always slow, and suggests that rapid recoveries are due to some other cause, very probably the functional nature of the lesion.

The view above referred to as to the existence of a psycho-motor speech-centre apart from the oral motor articulatory mechanism leads the author to place the seat of the mischief in hysterical mutism in the fibres connecting the two centres.

There is no need here for entering into further details of the many interesting discussions involved in the treatment of aphasia. We think that this book gives a very fair presentment of the subject, and that if read with Bastian's Lumleian lectures the student of aphasia would get a very complete acquaintance with our present knowledge of the subject. The two works admirably supplement each other, giving the arguments on both sides of many of the disputed points.

We conclude by commending this volume to our readers as deserving careful study; the author throughout gives the conclusions which he considers are established by theory and observed facts, but at the same time does justice to those who differ from him, is everywhere careful to point out how far theory is supported by pathological data, and is to be congratulated on having written a very useful and practical book.

